# Effect of Body Mass Index on the Prognosis of Liver Cirrhosis

**DOI:** 10.3389/fnut.2021.700132

**Published:** 2021-08-20

**Authors:** Yue Yin, Yiling Li, Lichun Shao, Shanshan Yuan, Bang Liu, Su Lin, Yida Yang, Shanhong Tang, Fanping Meng, Yunhai Wu, Yu Chen, Bimin Li, Qiang Zhu, Xingshun Qi

**Affiliations:** ^1^Liver Cirrhosis Study Group, Department of Gastroenterology, General Hospital of Northern Theater Command (formerly called General Hospital of Shenyang Military Area), Shenyang, China; ^2^Department of Gastroenterology, First Affiliated Hospital of China Medical University, Shenyang, China; ^3^Department of Gastroenterology, Air Force Hospital of Northern Theater Command, Shenyang, China; ^4^Department of Gastroenterology, Xi'an Central Hospital, Xi'an, China; ^5^Department of Hepatobiliary Disease, 900 Hospital of the Joint Logistics Team (formerly called Fuzhou General Hospital), Fuzhou, China; ^6^Liver Research Center, First Affiliated Hospital of Fujian Medical University, Fuzhou, China; ^7^State Key Laboratory for Diagnosis and Treatment of Infectious Diseases, Collaborative Innovation Center for Diagnosis and Treatment of Infectious Diseases, First Affiliated Hospital, College of Medicine, Zhejiang University, Hangzhou, China; ^8^Department of Gastroenterology, General Hospital of Western Theater Command, Chengdu, China; ^9^Department of Biological Therapy, Fifth Medical Center of PLA General Hospital, Beijing, China; ^10^Department of Critical Care Medicine, Sixth People's Hospital of Shenyang, Shenyang, China; ^11^Difficult and Complicated Liver Diseases and Artificial Liver Center, Beijing Youan Hospital, Capital Medical University, Beijing, China; ^12^Department of Gastroenterology, First Affiliated Hospital of Nanchang University, Nanchang, China; ^13^Department of Gastroenterology, Shandong Provincial Hospital Affiliated to Shandong First Medical University, Jinan, China

**Keywords:** body mass index, liver cirrhosis, obesity, prognosis, outcome

## Abstract

**Objective:** At present, the association of body mass index (BMI) with the prognosis of liver cirrhosis is controversial. Our retrospective study aimed to evaluate the impact of BMI on the outcome of liver cirrhosis.

**Methods:** In the first part, long-term death was evaluated in 436 patients with cirrhosis and without malignancy from our prospectively established single-center database. In the second part, in-hospital death was evaluated in 379 patients with cirrhosis and with acute gastrointestinal bleeding (AGIB) from our retrospective multicenter study. BMI was calculated and categorized as underweight (BMI <18.5 kg/m^2^), normal weight (18.5 ≤ BMI < 23.0 kg/m^2^), and overweight/obese (BMI ≥ 23.0 kg/m^2^).

**Results:** In the first part, Kaplan–Meier curve analyses demonstrated a significantly higher cumulative survival rate in the overweight/obese group than the normal weight group (*p* = 0.047). Cox regression analyses demonstrated that overweight/obesity was significantly associated with decreased long-term mortality compared with the normal weight group [hazard ratio (HR) = 0.635; 95% CI: 0.405–0.998; *p* = 0.049] but not an independent predictor after adjusting for age, gender, and Child–Pugh score (HR = 0.758; 95%CI: 0.479–1.199; *p* = 0.236). In the second part, Kaplan–Meier curve analyses demonstrated no significant difference in the cumulative survival rate between the overweight/obese and the normal weight groups (*p* = 0.094). Cox regression analyses also demonstrated that overweight/obesity was not significantly associated with in-hospital mortality compared with normal weight group (HR = 0.349; 95%CI: 0.096-1.269; *p* = 0.110). In both of the two parts, the Kaplan–Meier curve analyses demonstrated no significant difference in the cumulative survival rate between underweight and normal weight groups.

**Conclusion:** Overweight/obesity is modestly associated with long-term survival in patients with cirrhosis but not an independent prognostic predictor. There is little effect of overweight/obesity on the short-term survival of patients with cirrhosis and with AGIB.

## Introduction

Overweight/obesity, which is defined as excessive body fat accumulation, is a common public health problem (1). The global age-standardized prevalence of obesity defined by high body mass index (BMI) is increased from 3.2 to 10.8% in men and from 6.4 to 14.9% in women between 1975 and 2014 (2). Overweight/obesity is considered a risk factor for liver diseases (3, 4). Increased adipose tissues lead to triglyceride deposition in the liver, produce various transduction signals that alter lipid and glucose metabolisms, and then cause insulin resistance and increased release of free fatty acids, which are the causes of hepatic steatosis (5). Hepatic steatosis can contribute to lipid peroxidation and hepatic stellate cell activation, which further induce cellular injury and inflammation and accelerate the progression of liver fibrosis and cirrhosis (6, 7). However, the impact of BMI on outcomes in liver cirrhosis is still controversial. Some studies demonstrated that obesity was an independent risk factor for cirrhosis-related death or hospitalization (8). Other studies supported that the patients with cirrhosis and with obesity had a lower mortality than those without (9). Considering the controversy of the existing evidence, this study aimed to examine the effect of BMI on the prognosis of patients with liver cirrhosis.

## Methods

### Study Design

This retrospective study was carried out following the rules of the 1975 Declaration of Helsinki and approved by the Medical Ethical Committee of the General Hospital of Northern Theater Command with an approval number of Y (2021) 023. It was divided into two major parts. In both of the two parts, if the patients are lacking height and weight, they were excluded from the current study.

In the first part, we retrospectively selected patients with cirrhosis and without malignancy from our prospectively established database (10). Eligible patients should be consecutively admitted to our department and underwent an endoscopy and contrast-enhanced CT or MRI scans between December 2014 and December 2020. They were regularly followed *via* telephone or through outpatient visits and/or by reviewing medical records until February 2021. Death and the patients with liver transplantation during follow-up were recorded. Liver transplantation-free survival was the major endpoint of the first part. Patients who underwent liver transplantation were followed until the time point when the liver transplantation was performed.

In the second part, we retrospectively selected patients with cirrhosis and with acute gastrointestinal bleeding (AGIB) who received terlipressin and/or somatostatin/octreotide from our multicenter study (registration number: NCT03846180), which has been further updated after some publications (11–14). Notably, in this part, the eligible patients were consecutively admitted to 13 centers from 8 provinces or municipalities in China between January 2010 and December 2018, and patients who underwent transjugular intrahepatic portosystemic shunt, splenectomy, surgical shunt, or liver transplantation were excluded from the study. In-hospital death was the major endpoint of the second part.

### Diagnosis and Definitions

Liver cirrhosis was diagnosed based on clinical manifestations, laboratory tests, radiological examinations, and/or histological data. AGIB was defined as hematemesis, melena, and/or hematochezia within 5 days before admission (15).

BMI was calculated by dividing weight in kilograms by the square of height in meters (16). According to the WHO classification for Asian populations, all patients were categorized as underweight (BMI <18.5 kg/m^2^), normal weight (18.5 ≤ BMI < 23.0 kg/m^2^), and overweight/obese (BMI ≥ 23.0 kg/m^2^) (17).

### Statistical Analyses

First, continuous variables were described as mean ± SD and median (range), and categorical variables were described as frequency (percentage). The difference was compared using Mann–Whitney *U* test and Chi-squared test or Fisher's exact test. Second, survival probability curves were calculated by the Kaplan–Meier curve analyses and compared by the log-rank test. Third, univariate Cox regression analyses were performed to explore the association of BMI with mortality, and multivariable Cox regression analyses were performed by adjusting for age, gender, and Child–Pugh scores to identify whether BMI was an independent predictor of death. Hazard ratios (HRs) with 95% CIs were calculated. Fourth, time-dependent receiver operating characteristic (T-ROC) curve analyses were used to evaluate the performance of BMI for predicting death, and area under the curve (AUC) and concordance index (C-index) were calculated. A two-tailed *p* < 0.05 was considered statistically significant. All statistical analyses were performed by using SPSS version 26.0 (IBM Corp, Armonk, New York, USA) and R version 4.0.3 with packages survival, survminer, and timeROC (R Foundation for Statistical Computing, Vienna, Austria).

## Results

### First Part: Long-Term Outcomes of Patients With Cirrhosis

**Figure 1:**
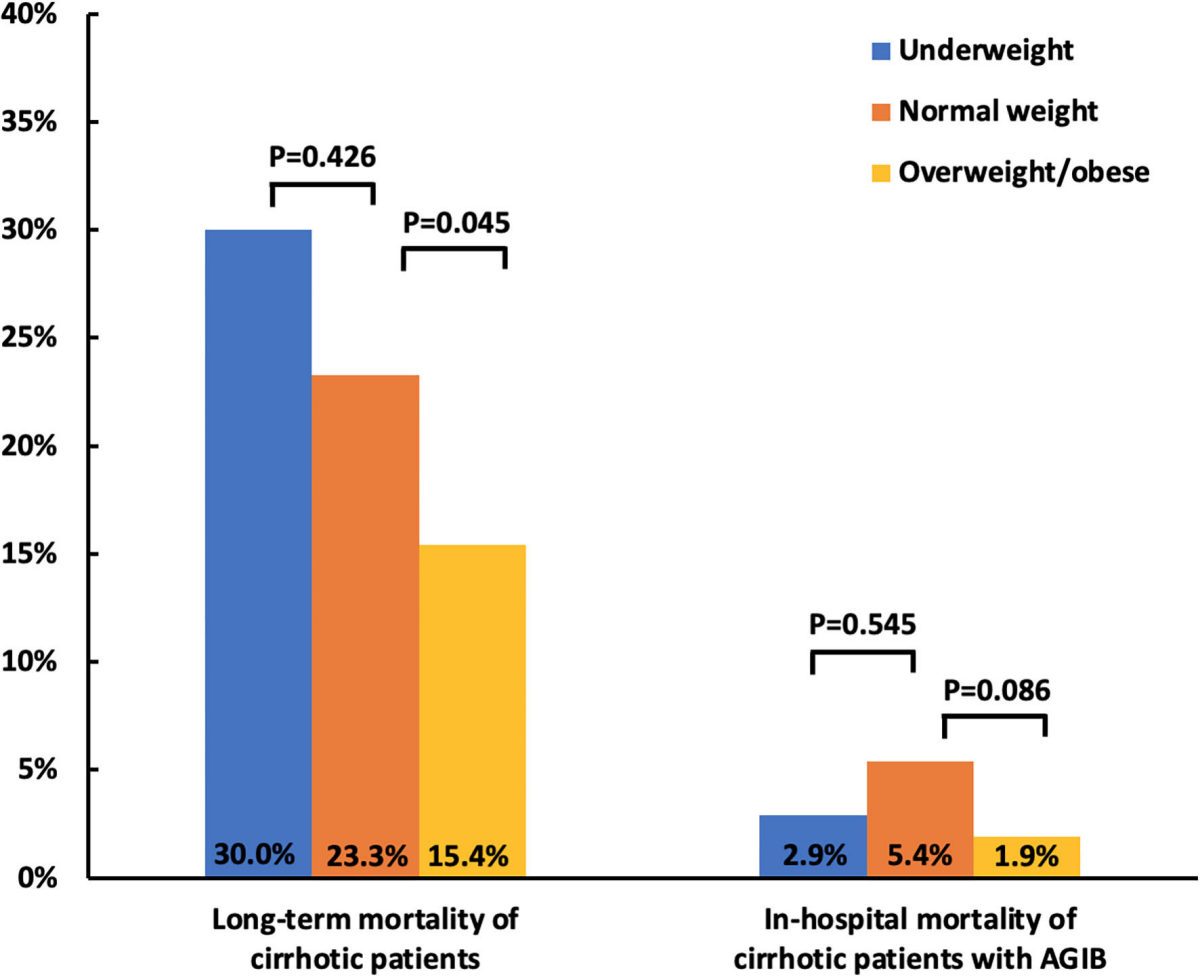
Bar charts showing the association of BMI with mortality in patients with cirrhosis. BMI, body mass index; AGIB, acute gastrointestinal bleeding.

Overall, 436 of 527 patients with cirrhosis registered in our prospective database had BMI data at their admissions and were included in the present study. Among them, 30 (6.9%) were underweight and 234 (53.7%) overweight/obese. The median BMI was 17.34 (range: 14.82–18.42 kg/m^2^) in underweight group, 21.02 (range: 18.52–22.95 kg/m^2^) in normal weight group, and 25.47 (range: 23.01–37.37 kg/m^2^) in overweight/obese group. During a median follow-up period of 2.28 (range: 0.03–5.59 years), 1 patient was lost to follow-up, 6 underwent liver transplantation, and 85 died. Among them, 62 (72.9%) patients died of liver diseases, 14 (16.5%) non-liver diseases, and 9 (10.6%) unknown causes. The mortality was 30% (9/30) in underweight group, 23.3% (40/172) in normal weight group, and 15.4% (36/234) in overweight/obese group (Figure 1).

**Figure 2:**
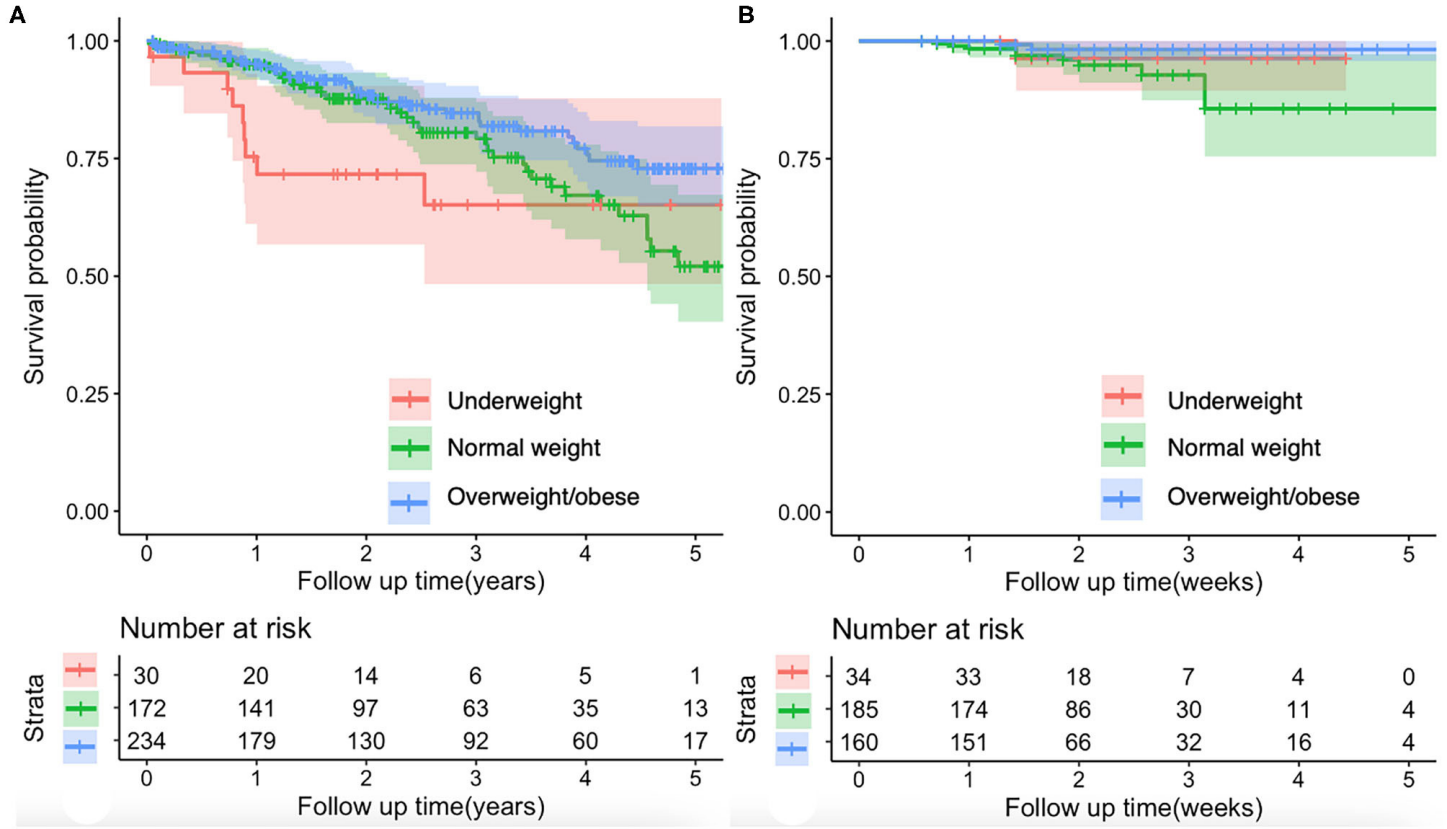
Kaplan–Meier curves showing the effect of BMI on the mortality of patients with cirrhosis. **(A)** Long-term mortality of patients with cirrhosis. There was no significant difference in the cumulative survival rate between normal weight and underweight groups (log-rank test, *p* = 0.190). The overweight/obese group had a significantly higher cumulative survival rate than the normal weight group (log-rank test, *p* = 0.047). **(B)** In-hospital mortality of patients with cirrhosis and with AGIB. There was no significant difference in the cumulative survival rate between normal weight and underweight groups (log-rank test, *p* = 0.491) or between normal weight and overweight/obese groups (log-rank test, *p* = 0.094). AGIB, acute gastrointestinal bleeding.

Compared with normal weight group, underweight group had significantly lower proportions of hepatitis C virus (HCV) (0 vs. 11.6%; *p* = 0.034) and international normalized ratio (INR) (1.31 ± 0.40 vs. 1.34 ± 0.26; *p* = 0.026) and higher platelet count (PLT) (163.10 ± 137.51 vs. 97.65 ± 66.90; *p* = 0.004) (Table 1). The Kaplan–Meier curve analyses demonstrated no significant difference in the cumulative survival rate between normal weight and underweight groups (*p* = 0.190) (Figure 2A). Univariate Cox regression analyses also demonstrated that underweight was not significantly associated with long-term mortality (HR = 1.617; 95%CI: 0.783–3.338; *p* = 0.194) (Supplementary Table 1).

**Table 1:** Baseline characteristics of patients with cirrhosis in the first part.

**Variables**	**No. pts**	**Normal weight group**	**No. pts**	**Underweight group**	*P*-value^*^	**No. pts**	**Overweight/obese group**	***P*-value^#^**
**Demographics**								
Age (years)	172	54.69 ± 10.51 55.51 (30.21–78.36)	30	56.95 ± 13.88 57.08 (20.57–81.09)	0.376	234	55.35 ± 10.39 55.13 (20.58–88.73)	0.407
Gender (male)	172	117 (68.0%)	30	15 (50.0%)	0.056	234	175 (74.8%)	0.134
BMI (kg/m^2^)	172	20.96 ± 1.24 21.02 (18.52–22.95)	30	17.31 ± 0.85 17.34 (14.82–18.42)	** <0.0001**	234	25.84 ± 2.36 25.47 (23.01–37.37)	** <0.0001**
**Comorbidities**								
Diabetes	172	24 (14.0%)	30	4 (13.3%)	0.928	234	34 (14.5%)	0.870
Hypertension	172	22 (12.8%)	30	2 (6.7%)	0.339	234	43 (18.4%)	0.129
**Etiology of liver cirrhosis**								
HBV	172	59 (34.3%)	30	11 (36.7%)	0.802	234	103 (44.0%)	**0.048**
HCV	172	20 (11.6%)	30	0	**0.034**	234	16 (6.8%)	0.093
Alcohol abuse	172	61 (35.5%)	30	11 (36.7%)	0.899	234	115 (49.1%)	**0.006**
**Laboratory tests**								
RBC (10^12^/L)	172	3.26 ± 0.84 3.24 (1.45–5.20)	30	3.17 ± 0.69 3.09 (1.78–4.56)	0.517	234	3.41 ± 0.86 3.43 (1.43–5.62)	0.148
WBC (10^9^/L)	172	4.46 ± 3.48 3.6 (0.7–23.1)	30	5.54 ± 3.92 4.35 (0.8–19.6)	0.085	234	4.11 ± 2.86 3.45 (1.0–30.4)	0.649
PLT (10^9^/L)	172	97.65 ± 66.90 77.5 (18–377)	30	163.10 ± 137.51 116 (39–646)	**0.004**	234	96.27 ± 59.60 81.5 (23–457)	0.577
TBIL (μmol/L)	172	27.34 ± 25.41 20.05 (5.2–172.1)	30	43.9 ± 52.9 15.5 (6.6–216.5)	0.870	234	29.56 ± 27.87 21.05 (4.9–215.3)	0.182
ALB (g/L)	171	31.94 ± 5.96 32.1 (17.2–46.0)	30	31.06 ± 5.44 31.15 (21.8–40.6)	0.450	233	32.99 ± 6.34 32.6 (14.2–50.6)	0.125
ALT (U/L)	172	33.45 ± 49.75 21.85 (6.58–590.00)	30	37.55 ± 58.50 21.38 (7.53–332.5)	0.887	234	41.98 ± 103.09 25.33 (4.23–1465.5)	0.090
GGT (U/L)	172	82.76 ± 140.76 35.57 (7.54–1081.31)	30	131.60 ± 232.46 58.64 (11.9–1283.02)	0.135	234	91.43 ± 183.08 41.88 (8.23–1779.18)	0.104
SCr (μmol/L)	170	67.37 ± 26.30 63.23 (14.80–267.63)	30	61.17 ± 19.36 57.45 (32.65–117.66)	0.173	230	65.38 ± 16.98 63.24 (16.50–131.91)	0.987
Na (mmol/L)	170	139.10 ± 2.83 139.0 (127.2–145.5)	30	136.90 ± 5.72 137.6 (118.0–146.7)	0.058	234	138.96 ± 2.95 139 (127–151)	0.727
INR	169	1.34 ± 0.26 1.29 (0.89–2.77)	30	1.31 ± 0.40 1.16 (0.93–2.43)	**0.026**	232	1.33 ± 0.27 1.25 (0.91–2.55)	0.334
Child-Pugh score	169	7.28 ± 1.80 7 (5–13)	30	8.0 ± 2.12 7 (5–12)	0.092	231	7.05 ± 1.88 7 (5–13)	0.124
Child-Pugh class A/B+C	169	68 (40.2%)/101 (59.8%)	30	9 (30.0%)/21 (70.0%)	0.289	231	106 (45.9%)/125 (54.1%)	0.260
MELD score	169	11.43 ± 3.57 10.50 (6.54–27.84)	30	12.45 ± 6.76 9.68 (6.43–30.03)	0.293	229	11.33 ± 4.09 10.04 (6.43–28.91)	0.232
**Decompensated events**								
Ascites	172	107 (62.2%)	30	22 (73.3%)	0.242	234	123 (52.6%)	0.053
HE	172	6 (3.5%)	30	0	0.376	234	5 (2.1 %)	0.407
AGIB	172	60 (34.9%)	30	9 (30.0%)	0.603	234	65 (27.8%)	0.125

* *P-value was compared between underweight and normal weight groups;*

# *p-value was compared between overweight/obese and normal weight groups. Bold numerals showed statistically significant*.

*No. Pts., numbers of patients; BMI, body mass index; HBV, hepatitis B virus; HCV, hepatitis C virus; RBC, red blood cell; WBC, white blood cell; PLT, platelet count; TBIL, total bilirubin; ALB, albumin; ALT, alanine aminotransferase; GGT, gamma-glutamyl transpeptidase; SCr, serum creatinine; Na, sodium; INR, international normalized ratio; MELD, model for end-stage liver disease; HE, hepatic encephalopathy; AGIB, acute gastrointestinal bleeding*.

Compared with normal weight group, overweight/obese group had significantly higher proportions of hepatitis B virus (HBV) (44.0 vs. 34.3%; *p* = 0.048) and alcohol abuse (49.1 vs. 35.5%; *p* = 0.006) (Table 1) and lower mortality (15.4 vs. 23.3%; *p* = 0.045) (Figure 1). The Kaplan-Meier curve analyses demonstrated that overweight/obese group had a significantly higher cumulative survival rate than normal weight group (*p* = 0.047) (Figure 2A). Univariate Cox regression analyses demonstrated that overweight/obesity was significantly associated with decreased long-term mortality (HR = 0.635; 95%CI: 0.405–0.998; *p* = 0.049). After adjusting for age, gender, and Child–Pugh score, overweight/obesity was not an independent predictor of decreased long-term mortality (HR = 0.758; 95%CI: 0.479–1.199; *p* = 0.236) (Supplementary Table 1).

**Figure 3:**
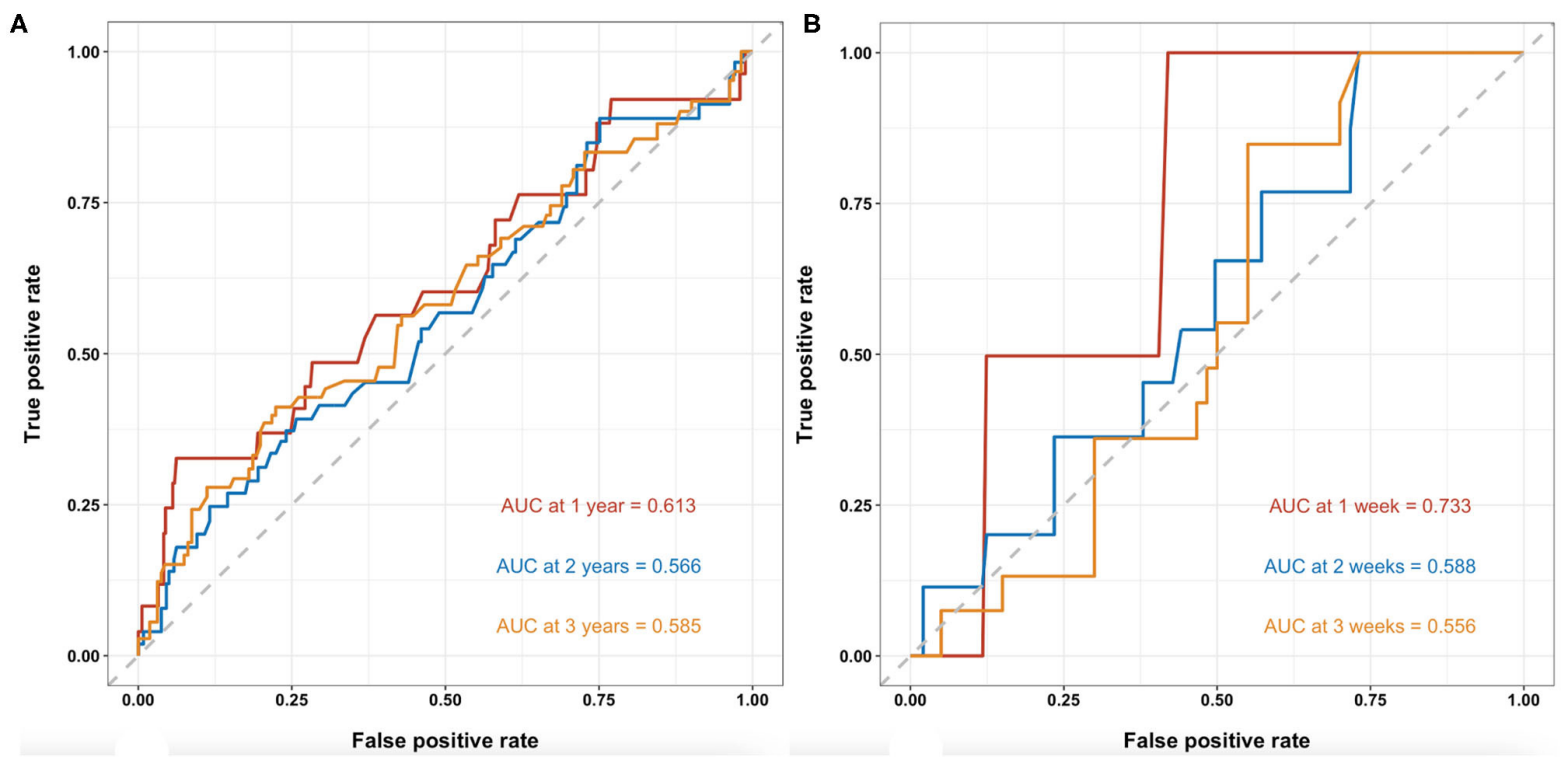
T-ROC curves of BMI for the predictability of mortality in patients with cirrhosis. **(A)**. Long-term mortality of patients with cirrhosis at 1-, 2-, and 3-year during follow-up. The AUCs were 0.613 (95%CI: 0.487–0.740), 0.566 (95%CI: 0.472–0.659), and 0.585 (95%CI: 0.495–0.675), respectively. **(B)** In-hospital mortality of patients with cirrhosis with AGIB at 1-, 2-, and 3-week during hospitalization. The AUCs were 0.733 (95%CI: 0.527–0.938), 0.588 (95%CI: 0.422–0.755), and 0.556 (95%CI: 0.410–0.702), respectively. T-ROC, time-dependent receiver operating characteristic; BMI, body mass index; AUC, area under the curve.

Time-dependent receiver operating characteristic analyses of BMI for predicting long-term mortality of patients with cirrhosis are shown in Figure 3A. The AUCs at 1-, 2-, and 3-year during follow up were 0.613 (95%CI: 0.487–0.740), 0.566 (95%CI: 0.472–0.659), and 0.585 (95%CI: 0.495–0.675), respectively. C-index was 0.568 (95% CI: 0.497–0.639).

### Second Part: In-hospital Outcomes of Patients With Cirrhosis and With AGIB

Overall, 379 of 1,582 patients with cirrhosis and with AGIB recorded in our multicenter study had BMI data at their admissions and were included in the present study. Among them, 34 (9.0%) were underweight and 160 (42.2%) overweight/obese. The median BMI was 17.70 (range: 16.33–18.49 kg/m^2^)in underweight group, 21.09 (range: 18.56–22.99 kg/m^2^) in normal weight group, and 25.15 (range: 23.03–43.94 kg/m^2^) in overweight/obese group. During a median hospitalization period of 13 (range: 4–48) days, 14 patients died. Among them 11 (78.6%) patients died of liver diseases and 3 (21.4%) died of non-liver diseases. In-hospital mortality was 2.9% (1/34) in underweight group, 5.4% (10/185) in normal weight group, and 1.9% (3/160) in overweight/obese group (Figure 1).

Compared with normal weight group, underweight group had significantly lower proportion of men (47.1 vs. 75.1%; *p* = 0.001) and higher PLT (113.32 ± 81.32 vs. 86.69 ± 65.13; *p* = 0.004) (Table 2). The Kaplan–Meier curve analyses demonstrated no significant difference in the cumulative survival rate between normal weight and underweight groups (*p* = 0.491) (Figure 2B). Univariate Cox regression analyses also demonstrated that underweight was not significantly associated with in-hospital mortality (HR = 0.494; 95%CI: 0.063–3.866; *p* = 0.502) (Supplementary Table 1).

**Table 2:** Baseline characteristics of patients with cirrhosis and with AGIB in the second part.

**Variables**	**No. Pts**	**Normal weight group**	**No. Pts**	**Underweight group**	*P*-value^*^	**No. Pts**	**Overweight/obese group**	***P*-value^#^**
**Demographics**								
Age (years)	185	55.19 ± 12.46 54.62 (22.98–82.01)	34	57.35 ± 15.78 55.64 (23–88.21)	0.458	160	55.42 ± 12.53 54.26 (25.44–86.39)	0.851
Gender (male)	185	139 (75.1%)	34	16 (47.1%)	**0.001**	160	131 (81.9%)	0.130
BMI (kg/m^2^)	185	21.05 ± 1.34 21.09 (18.56–22.99)	34	17.59 ± 0.60 17.70 (16.33–18.49)	** <0.0001**	160	26.03 ± 2.93 25.16 (23.03–43.94)	** <0.0001**
**Comorbidities**								
Diabetes	185	27 (14.6%)	34	3 (8.8%)	0.368	160	33 (20.6%)	0.141
Hypertension	185	23 (12.4%)	34	5 (14.7%)	0.715	160	22 (13.8%)	0.717
**Etiology of liver cirrhosis**								
HBV	185	116 (62.7%)	34	17 (50.0%)	0.163	160	95 (59.4%)	0.527
HCV	185	7 (3.8%)	34	2 (5.9%)	0.571	160	9 (5.6%)	0.417
Alcohol abuse	185	43 (23.2%)	34	4 (11.8%)	0.134	160	40 (25%)	0.703
**Laboratory tests**								
RBC (10^12^/L)	185	2.92 ± 0.86 2.86 (1.15–5.14)	34	2.92 ± 0.83 2.82 (1.33–4.68)	0.986	160	2.98 ± 0.76 2.85 (1.34–5.26)	0.422
WBC (10^9^/L)	185	6.15 ± 4.03 5.39 (1.15–23.4)	34	6.52 ± 3.68 6.51 (1.3–17.1)	0.328	160	6.76 ± 4.59 5.89 (1.4–31.04)	0.087
PLT (10^9^/L)	185	86.69 ± 65.13 68 (2–476)	34	113.32 ± 81.32 97.5 (21–473)	**0.004**	160	85.04 ± 42.19 78 (5–201)	0.197
TBIL (μmol/L)	185	50.02 ± 73.01 24.73 (3.9–518)	34	33.60 ± 40.46 25.4 (4–244.6)	0.434	160	43.36 ± 76.41 24.2 (6.4–720.9)	0.509
ALB (g/L)	185	30.51 ± 5.92 31 (11.0–46.8)	34	30.52 ± 6.27 30.45 (10.1–44.6)	0.893	160	29.98 ± 6.12 30 (14.6–49.8)	0.313
ALT (U/L)	185	67.77 ± 238.96 30.0 (6.3–2651)	34	41.84 ± 33.60 31.2 (8.62–140)	0.611	160	60.49 ± 141.16 33.15 (6.42–1575)	**0.039**
GGT (U/L)	185	124.26 ± 223.83 46.9 (6–1958)	34	117.26 ± 135.69 52.5 (6–531)	0.829	160	113.92 ± 221.86 45.2 (7–2145)	0.688
SCr (μmol/L)	185	70.05 ± 22.73 66.7 (25–202.2)	34	72.6 ± 46.17 64.2 (27–305)	0.338	160	75.36 ± 27.03 70.05 (24.9–223.3)	**0.044**
Na (mmol/L)	185	137.51 ± 5.10 138.0 (115.6–157)	34	136.61 ± 5.71 137.3 (121.8–148)	0.401	159	137.49 ± 4.39 138 (119.7–147.5)	0.965
INR	182	1.39 ± 0.31 1.33 (0.92–3.04)	34	1.33 ± 0.26 1.27 (1.07–2.48)	0.189	159	1.40 ± 0.29 1.32 (0.98–2.77)	0.684
Child-Pugh score	182	7.79 ± 1.77 8 (5–12)	34	7.82 ± 1.62 7.5 (5–12)	0.917	159	7.64 ± 1.96 7 (5–15)	0.235
Child-Pugh class A/B+C	182	49 (26.9%)/133 (73.1%)	34	7 (20.6%)/27 (79.4%)	0.439	159	48 (30.2%)/111 (69.8%)	0.505
MELD score	182	13.86 ± 5.71 12.04 (6.43–32.59)	34	13.54 ± 5.51 11.73 (7.5–28.72)	0.867	159	13.61 ± 5.55 12.08 (6.9–37.59)	0.816
**Decompensated events**								
Ascites	185	114 (61.6%)	34	23 (67.6%)	0.505	160	89 (55.6%)	0.259
HE	185	10 (5.4%)	34	3 (8.8%)	0.438	160	6 (3.8%)	0.466

* *P-value was compared between underweight and normal weight groups;*

# *p-value was compared between overweight/obese and normal weight groups. Bold numerals showed statistically significant. AGIB, acute gastrointestinal bleeding; No. Pts., numbers of patients; BMI, body mass index; HBV, hepatitis B virus; HCV, hepatitis C virus; RBC, red blood cell; WBC, white blood cell; PLT, platelet count; TBIL, total bilirubin; ALB, albumin; ALT, alanine aminotransferase; GGT, gamma-glutamyl transpeptidase; SCr, serum creatinine; Na, sodium; INR, international normalized ratio; MELD, a model for end-stage liver disease; HE, hepatic encephalopathy*.

Compared with normal weight group, overweight/obese group had significantly lower alanine aminotransferase (ALT) (60.49 ± 141.16 vs. 67.77 ± 238.96; *p* = 0.039) and higher serum creatinine (SCr) (75.36 ± 27.03 vs. 70.05 ± 22.73; *p* = 0.044) (Table 2). The Kaplan–Meier curve analyses demonstrated no significant difference in the cumulative survival rate between normal weight and overweight/obese groups (*p* = 0.094) (Figure 2B). Univariate Cox regression analyses also demonstrated that overweight/obesity was not significantly associated with in-hospital mortality (HR = 0.349; 95%CI: 0.096–1.269; *p* = 0.110) (Supplementary Table 1).

Time-dependent receiver operating characteristic analyses of BMI for predicting in-hospital mortality of patients with cirrhosis and with AGIB are shown in Figure 3B. The AUCs at 1-, 2-, and 3-week during hospitalizations were 0.733 (95%CI: 0.527–0.938), 0.588 (95%CI: 0.422–0.755), and 0.556 (95%CI: 0.410–0.702), respectively. C-index was 0.610 (95% CI: 0.477–0.742).

## Discussion

The first objective of the present work was to explore the relationship of BMI with the long-term prognosis of patients with cirrhosis. We found that overweight/obesity was inversely associated with long-term mortality of patients with liver cirrhosis. This finding supported the “obesity paradox” that overweight/obese patients could have superior survival. It was first proposed by Fleischmann et al. (18) in patients undergoing hemodialysis (18) and further validated in subjects with chronic diseases, such as cardiovascular diseases, hypertension, and diabetes (19–22).

The pathophysiology of the “obesity paradox” remains to be elucidated, and there are some underlying explanations (Supplementary Figure 1). First, fat storage in overweight/obese patients may protect the balance of muscle protein catabolism in chronic wasting diseases (23). Body protein is crucial for survival because it can maintain cell function and support cell architecture (24). Muscle protein metabolism is preserved in patients with obesity and with chronic cardiac failure, indicating better outcomes but increased in patients without obesity (25). Similarly, sarcopenia, which is mainly caused by increased muscle protein metabolism (26, 27), is associated with lower BMI in patients with liver cirrhosis (28), and further leads to higher mortality (29). Second, adipose tissue, which has been recognized as an endocrine organ, can secrete diverse adipokines (30, 31). Adiponectin, an anti-inflammatory adipokine, can inhibit the proliferation and activation of hepatic stellate cells, which produce extracellular matrix proteins in the case of liver injury and promote the occurrence of liver fibrosis (32). Leptin, another adipokine, can prevent ectopic lipid accumulation in non-adipose tissues, augment immune response, and improve bacterial clearance and survival in animal models (33, 34). Both of which are increased in overweight/obese patients with liver cirrhosis, probably improving the outcomes of the patients (35). Third, patients with cirrhosis and with hepatic edema have systemic vasodilation and underfilled arteries, decreasing effective circulatory blood volume and activating cardiac sympathetic nervous system (SNS) and renin-angiotensin-aldosterone system (RAAS) which can stimulate sodium and water retention. Prolonged sodium and water retention will cause hyponatremia and pulmonary edema and increase cardiac afterload (36, 37). Overweight/obesity can alleviate the activities of cardiac SNS and RAAS, thereby inhibiting hyperdynamic circulation and conferring survival benefits (38). Fourth, overweight/obese patients are more likely to receive medical interventions, including antihypertensive drugs for decreasing systolic blood pressure, which can make short-term hemodynamic status more stable (39, 40), and statins for treating hyperlipidemia (41), which can have a favorable impact on outcomes of cirrhosis and portal hypertension (42).

Age, gender, and body function may interact with the relationship between BMI and prognosis (43, 44). Accordingly, this study adjusted some potential confounding factors, including age, gender, and Child–Pugh score, in multivariable Cox regression analysis. By comparison, the previous study by Karagozian et al. (9) selected patients with cirrhosis from the National Inpatient Sample database, which was lacking laboratory data, such as hepatic function (9). Thus, these results should be more reliable. This study found that BMI was not an independent risk factor of decreased long-term mortality, indicating that the prognostic impact of BMI might not be as strong as Child–Pugh score. This finding can be explained by the hypothesis of “reverse causation” that overweight/obesity may not be a cause for a better outcome, but a consequence (45–47). Another explanation is that BMI is convenient but unable to comprehensively measure body composition (2, 48), such as muscle and subcutaneous and visceral adipose tissue and their specific distributions in the body (49). Besides, body weight may be masked by fluid retention resulting from ascites in patients with cirrhosis, inaccurately or falsely evaluating the prognostic impact of BMI.

This study demonstrated that BMI was not significantly associated with in-hospital outcomes of patients with cirrhosis and with AGIB, which is consistent with the previous study regarding the association of obesity with in-hospital mortality of patients with non-variceal gastrointestinal bleeding (50). This may be explained by the complexity of evaluating the outcomes of acute injuries, which should not be attributed to the effect of body weight alone. Fatal injuries brought by decompensated events are far beyond the potential benefits of overweight/obesity.

There were some limitations in this study. First, we did not obtain the dynamic changes of BMI during follow-up. Second, we did not have an external validation cohort to verify the present findings. Third, BMI data were missing in a proportion of our AGIB patients, probably producing a selection bias. Fourth, the interventions used during the hospitalization and follow-up, which might be beneficial for the outcomes, were not available. Fifth, we did not evaluate the specific values of muscle mass or the distribution of adipose tissue due to the absence of dual-energy X-ray absorptiometry, waist circumference, and waist to hip ratio.

In conclusion, there is a modest association of overweight/obesity with decreased long-term mortality of patients with cirrhosis. However, BMI cannot act as an independent prognostic predictor of liver cirrhosis. In the future, prospective large-scale studies should be attempted to combine BMI with other indicators involved in measuring muscle mass and adipose tissue to more precisely predict the clinical outcomes of liver cirrhosis.

## Data Availability Statement

The raw data supporting the conclusions of this article will be made available by the authors, without undue reservation.

## Ethics Statement

The studies involving human participants were reviewed and approved by Medical Ethical Committee of the General Hospital of Northern Theater Command. Written informed consent for participation was not required for this study in accordance with the national legislation and the institutional requirements.

## Author Contributions

XQ: conceptualization and supervision. YYi and XQ: methodology, formal analysis, visualization, and writing—original draft. YL, LS, SY, BL, SL, YYa, ST, FM, YW, YC, BL, QZ, and XQ: resource. YYi, YL, LS, SY, BL, SL, YYa, ST, FM, YW, YC, BL, QZ, and XQ: data curation and writing—review and editing. All authors have made an intellectual contribution to the manuscript and approved the submission.

## Conflict of Interest

The authors declare that the research was conducted in the absence of any commercial or financial relationships that could be construed as a potential conflict of interest.

## Publisher's Note

All claims expressed in this article are solely those of the authors and do not necessarily represent those of their affiliated organizations, or those of the publisher, the editors and the reviewers. Any product that may be evaluated in this article, or claim that may be made by its manufacturer, is not guaranteed or endorsed by the publisher.

## Supplementary Material

Supplementary Figure 1 Schematic diagram showing the underlying explanations of obesity paradox. SNS, sympathetic nervous system; RAAS, renin-angiotensin-aldosterone system.

## Abbreviations

BMI: body mass index
AGIB: acute gastrointestinal bleeding
HR: hazard ratio
T-ROC: time-dependent receiver operating characteristic
AUC: area under the curve
C-index: concordance index
HCV: hepatitis C virus
INR: international normalized ratio
PLT: platelet count
HBV: hepatitis B virus
ALT: alanine aminotransferase
SCr: serum creatinine
SNS: sympathetic nervous system
RAAS: renin-angiotensin-aldosterone system.
