# Supplementary material for: Effect of Body Mass Index on the Prognosis of Liver Cirrhosis
Source: Front Nutr. 2021 Aug 20;8:700132. doi: 10.3389/fnut.2021.700132 (PMC8417598; doi:10.3389/fnut.2021.700132)
Supplement: Supplementary file 1 [file Table_1.DOCX]

| **Supplementary table 1. Cox regression analyses regarding effect of BMI on mortality in cirrhotic patients** | | | | | | | |
| --- | --- | --- | --- | --- | --- | --- | --- |
| **Variables** | **Univariate analyses** | | |  | **Multivariable analyses** | | |
|  | **HR** | **95% CI** | **P value** |  | **HR** | **95% CI** | **P value** |
| ***Long-term mortality in normal weight and underweight groups.*** | | | | | | | |
| Age* | 1.024 | 0.998-1.051 | 0.076 |  |  |  |  |
| Male versus female^#^ | 1.469 | 0.779-2.772 | 0.235 |  |  |  |  |
| Underweight versus normal weight^#^ | 1.617 | 0.783-3.338 | 0.194 |  |  |  |  |
| Child-Pugh score* | 1.391 | 1.204-1.608 | **<0.0001** |  |  |  |  |
| ***Long-term mortality in normal weight and overweight/obese groups.*** | | | | | | | |
| Age* | 1.022 | 1.001-1.045 | **0.044** |  | 1.034 | 1.011-1.058 | **0.004** |
| Male versus female^#^ | 1.611 | 0.928-2.797 | 0.090 |  | 1.558 | 0.887-2.734 | 0.123 |
| Overweight/obesity versus normal weight^#^ | 0.635 | 0.405-0.998 | **0.049** |  | 0.758 | 0.479-1.199 | 0.236 |
| Child-Pugh score* | 1.370 | 1.226-1.531 | **<0.0001** |  | 1.389 | 1.230-1.569 | **<0.0001** |
| ***Short-term mortality in normal weight and underweight groups.*** | | | | | | | |
| Age* | 1.038 | 0.990-1.088 | 0.121 |  |  |  |  |
| Male versus female^#^ | 1.080 | 0.286-4.078 | 0.910 |  |  |  |  |
| Underweight versus normal weight^#^ | 0.494 | 0.063-3.866 | 0.502 |  |  |  |  |
| Child-Pugh score* | 1.463 | 1.053-2.034 | **0.023** |  |  |  |  |
| ***Short-term mortality in normal weight and overweight/obese groups.*** | | | | | | | |
| Age* | 1.038 | 0.991-1.088 | 0.112 |  |  |  |  |
| Male versus female^#^ | 0.840 | 0.226-3.115 | 0.794 |  |  |  |  |
| Overweight/obesity versus normal weight^#^ | 0.349 | 0.096-1.269 | 0.110 |  |  |  |  |
| Child-Pugh score* | 1.393 | 1.048-1.852 | **0.023** |  |  |  |  |
| **Abbreviations:** BMI, body mass index; HR, hazard ratio; CI, confidence interval; AGIB, acute gastrointestinal bleeding.  **Notes:** *The variable was continuous; ^#^The variable was categorical. | | | | | | | |
