# Supplementary figures and images for: Effect of Body Mass Index on the Prognosis of Liver Cirrhosis
Source: Front Nutr. 2021 Aug 20;8:700132. doi: 10.3389/fnut.2021.700132 (PMC8417598; doi:10.3389/fnut.2021.700132)

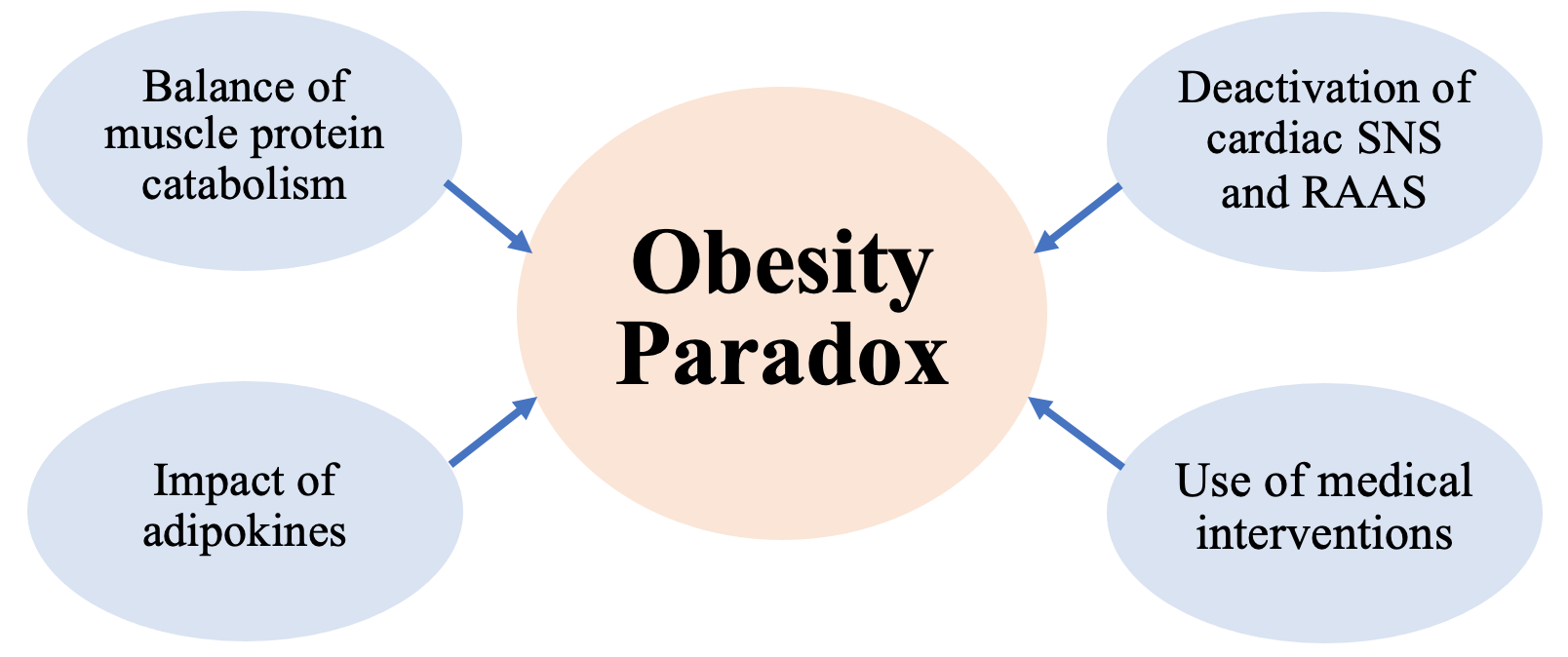

Supplement: Supplementary Figure 1 — Schematic diagram showing the underlying explanations of obesity paradox. SNS, sympathetic nervous system; RAAS, renin-angiotensin-aldosterone system. [file Image_1.JPEG]
